# The role of parametric linkage methods in complex trait analyses using microsatellites

**DOI:** 10.1186/1471-2156-6-S1-S48

**Published:** 2005-12-30

**Authors:** Michael D Badzioch, Ellen L Goode, Gail P Jarvik

**Affiliations:** 1 Department of Medicine, Division of Medical Genetics, University of Washington, Seattle, Washington, USA; 2 Department of Health Sciences Research, Mayo Clinic College of Medicine, Rochester, MN 55905 USA; 3 Departments of Epidemiology and Genome Sciences, University of Washington, Seattle, Washingon, USA

## Abstract

Many investigators of complexly inherited familial traits bypass classical segregation analysis to perform model-free genome-wide linkage scans. Because model-based or parametric linkage analysis may be the most powerful means to localize genes when a model can be approximated, model-free statistics may result in a loss of power to detect linkage. We performed limited segregation analyses on the electrophysiological measurements that have been collected for the Collaborative Study on the Genetics of Alcoholism. The resulting models are used in whole-genome scans. Four genomic regions provided a model-based LOD > 2 and only 3 of these were detected (*p *< 0.05) by a model-free approach. We conclude that parametric methods, using even over-simplified models of complex phenotypes, may complement nonparametric methods and decrease false positives.

## Background

Although model-based or parametric linkage analysis on extended families is generally considered the most powerful means to localize genes when a model can be approximated, the requirement for reasonable model parameter values is often perceived to be unattainable for complex traits. As a result, the potential advantages of the method are frequently passed over in favor of "model-free", nonparametric statistics that may be less powerful [1,2]. The rejection of parametric methods increases the possibility of missing linkage signals. It is generally recognized that failure to detect quantitative trait loci (QTLs) on an initial scan is more problematic than false positives. The latter should be excluded by follow-up analyses, but a false negative region may not be pursued. Thus, consideration of how to avoid false negatives is warranted. To date, five linkage studies have been performed on the electrophysiological measurements (electroencephalogram, EEG and event-related potential, ERP) that have been collected for the Collaborative Study on the Genetics of Alcoholism (COGA) dataset [3-7]. All of these linkage studies are based on identity-by-descent (IBD) allele sharing status, a "model-free" approach; four used variance decomposition as incorporated in SOLAR [3-6] and one used a regression approach [7]. The purpose of the current paper is to examine the utility of modeling the familial transmission of EEG and ERP phenotypes followed by 3-point, model-based linkage analysis of these traits versus model-free methods.

## Methods

### Phenotypes

We evaluated 13 quantitative traits representing neurological function in the COGA dataset (143 families). These included one EEG phenotype, ecb21, and 3 sets of 4 related ERP phenotypes (1 = FP1, 2 = FZ, 3 = CZ, and 4 = PZ channels), ttth1, tth2, tth3, and ttth4; ttdt1, ttd2, ttd3, and ttdt4; and ntt1, ntt2, ntt3, and ntt4. Linear regression was used to adjust for the effects of age and sex. The variable ecb21 was adjusted for sex, age, age^2^, and age^3^, and the remaining variables were adjusted for sex, age, and age^2^; all effects were highly significant. Additionally, the regression residuals for each of the 143 families were re-centered on zero to remove any family-specific effects on the mean.

### Modeling

Complex segregation analyses (CSA), using PAP v. 5, was used to estimate co-dominant (no overdominance) and dominant mixed models for each adjusted trait [8,9]. Multiple trials with random starting parameter values ensured maximum likelihood convergence for each model.

### Linkage analyses

Using the more parsimonious CSA model for each trait, we performed a two-marker genomic scan using LINKMAP [10,11]. Thus, we used each marker twice, except the p and q terminal markers of each chromosome, which were used once. Sixty likelihoods were calculated for each 2-marker set, 20 from theta = 0.5 to theta = 0 relative to marker 1, 20 between markers, and 20 from theta = 0 (relative to marker 2) to theta = 0.5. The possibility of linkage heterogeneity was evaluated using heterogeneity LOD scores (hLOD) using HOMOG [12]. A 2-point model-free scan was also done for each of the 13 phenotypes using the computer program MERLIN [13]. This program calculates an allele-sharing statistic, the Kong and Cox LOD (KC-LOD), and its statistical significance, p(KC). Eleven pedigrees too large for MERLIN analysis were trimmed for MERLIN analysis but not for the LINKAGE analysis. However, of the 44 individuals trimmed, only 5 had any measured phenotype. Regions for which a LOD score > 2 was detected were further evaluated by multipoint model-free analysis for comparison purposes.

## Results

For 9 of 13 traits the co-dominant models either resulted in over-dominance or failed to converge; these were not considered further. For the remaining traits (ecb21, ntth1, ntth3, and ttdt2) the dominant model was found to be more parsimonious (Table 1). In no case was the resulting polygenic inheritance greater than 0.0001. In the 13 2-point scans MERLIN found 29 linkages with *p *< 0.05 (data not shown). Using the Mendelian dominant genetic model for each trait, the parametric 2-marker genome scans detected 4 regions with a LOD score of at least 2.0 for 3 of the 13 traits (Table 2). None of these linkages showed significant heterogeneity. One of these regions, on chromosome 4, was not detected with a *p *< 0.05 using the model-free approach.

## Discussion

Model-based LOD score linkage has proven effective in localizing genes associated with numerous disease-related traits which, generally, exhibit Mendelian patterns of inheritance and for which the parameters (mode of transmission, gene frequency, and penetrance or quantitative effect size) have been estimated. However, the models available in current linkage software are overly simple for complex traits and the utility of model-based methods under these limitations is unclear. An incorrect model may lead to loss of power in the presence of true linkage as well as an overestimation of recombination [14]. Several strategies have been suggested to overcome these limitations of model-based linkage in complex traits. When the 'true' genetic model is unknown, maximizing the LOD score over several modes of inheritance, usually a dominant one and a recessive one, has been proposed [14]. Additionally, nonparametric or "model-free" linkage analysis methods are often used. Model-free methods, however, often put constraints on pedigree size and, overall, may have less power than model-based analyses, even for complex traits [15]. Thus, if the true model can be approximated, a model-based approach is desirable, especially in a genome scan, where exact specification of recombination values is of secondary concern.

Our findings indicate that model-based linkage of complex traits may add information not furnished by nonparametric analyses. Our two-marker parametric linkage results suggest four regions with LOD > 2 for three traits. The multipoint nonparametric analysis detected three of these regions with a *p *< 0.05 but did not detect the chromosome 4 region at this probability level. Two of the regions identified with LOD > 2 using parametric linkage appear to have been previously detected in published analyses of these data, while two others were not. Both previously detected regions were found by the program, SOLAR, which calculates a likelihood ratio derived LOD score by comparing a model in which the additive genetic variance at a specified map position is compared to one in which this component is set to zero. The chromosome 3 region (max LOD = 2.01 for ttdtla), near D3S2406-GATA128C02-D3S2459, is the same location in which Porjesz et al. [6], obtained a LOD of 2.59 for the N1 component, P4 lead trait. This was a different ERP component and lead compared to the P3-FP1 measurement giving our chromosome 3 linkage. Although Porjesz et al. [6] included the P3-FP1 phenotype in their study, they report linkage only on chromosome 5 (LOD 2.64). We did not have the N1 component available.

On chromosome 4 we obtained a LOD of 2.08 for ecb21 with D4S1558-D4S2361 whereas Williams et al. [4] found a peak multipoint LOD score of 1.51 (bivariate LOD = 2.65) at D4S1628 for the ERP phenotype, P3-CZ. Although the markers D4S2361 and D4S1628 are separated by 27 cM (Marshfield sex-averaged map), these two findings may represent distinct signals. Using Markov chain Monte Carlo (MCMC) methods, Sieh et al. [16] obtained a strong linkage signal for ecb21 at GABRB1 (51.4 cM on the Kosambi map) with a separate, weaker linkage at D4S1558. Using variance-components linkage analysis Lin et al. [17] obtained a LOD peak of 1.96 at 108 cM with ecb21 that apparently shifted to 95 cM (adjacent to D4S1559), LOD 4.38, when ALDX1 was added to create a bivariate trait. The scores on chromosome 9 for ttth1 (LOD = 2.30, GATA175H06-D9S925) and on chromosome 12 for ecb21 (LOD = 2.61, D12S1090-D12S390 at 9p22.2 and LOD = 2.17, D12S390-D12S398 at 12q13.13) appear to reference novel loci for EEG and ERP phenotypes. Indeed, the two adjacent chromosome 12 scores were the highest in our 13 genome scans, the largest associated with a *p*-value of 0.0005. Some support for linkage on this chromosome is suggested by the MERLIN results in that, of the 4 regions analyzed by multipoint linkage, chromosome 12 gave the highest KC-LOD, 0.9, and the smallest p(KC), 0.02.

Although, in this instance, our procedure resulted in the identification of a possibly 'real' linkage that was missed by standard nonparametric analysis, the use of nonparametric linkage methods shouldn't be viewed as inferior nor should our CSA be viewed as sufficient. The nonparametric linkage tests found candidate loci that our model-based procedure did not and our CSA was limited to a single major gene and forced convergence within a restricted sample space. Complex traits with multiple genetic and environmental effects will often result in no reasonable model. We found linkages for only four of thirteen modeled traits. Failure to detect linkage may have been due to unaccounted sources of familial correlation (e.g., environmental) or to modeling a single major gene when one did not exist. Also, when linkage is found using an overly-simple model, sensitivity testing to evaluate parameter values, e.g., marker allele frequencies and QTL penetrance/quantitative effect, can assess potential misspecification. However, overall, we recommend obtaining maximum likelihood genetic models from CSA whenever possible; by definition no 'truer' trait models can be obtained, given the single major gene restraint conditions under which we modeled.

## Conclusion

Our results indicate that model-based linkage procedures using simple models from CSA may detect candidate loci for complex traits that are not revealed by commonly used model-free techniques. Parametric methods that allow more complex modeling, such as MCMC methods, are being implemented [16,18,19]. However, the older model-based methods have been shown to complement MCMC approaches in complex trait linkage analyses and, in fact, may be advantageous for initial screening [19]. Until procedures for generating and utilizing complex trait linkage models are more widely available, parametric analyses under simpler models and nonparametric methods might be better used in a complementary manner.

## Abbreviations

COGA: Collaborative Study on the Genetics of Alcoholism

CSA: Complex segregation analysis

EEG: Electroencephalogram

ERP: Event-related potential

GAW: Genetic Analysis Workshop

HLOD: Heterogeneity LOD

IBD: Identity by descent

KC-LOD: Kong-Cox LOD

MCMC: Markov chain Monte Carlo

QTL: Quantitative trait locus

## Authors' contributions

MDB formulated the study question, did the analyses, and wrote the manuscript; ELG and GPJ provided critical input on the analyses and manuscript.

## Figures and Tables

**Table 1:** Genetic models from PAP segregation analysis for adjusted traits.

**Adjusted trait**	**Model**	**m(AA)^a^**	**m(Aa)**	**m(aa)**	**SD**	**f(a)^b^**	**-2lnLIKE**	**χ^2d^**	***p*-value^d^**
ecb21	Dom	-0.44	[-0.44]^c^	10.53	3.56	0.231	2625.5	37.5	0.000000
ecb21	CoDom	-0.12	14.31	16.73	3.95	0.008	2663.0		

ntth1	Dom	-0.02	[-0.02]	1.12	0.37	0.143	815.9	21.2	0.000004
ntth1	CoDom	-0.01	1.03	3.50	0.38	0.008	837.1		
ntth2	Dom	-0.05	[-0.05]	1.35	0.49	0.202	N/A^e^		
ntth3	Dom	-0.03	[-0.03]	1.38	0.52	0.167	1429.5	13.54	0.000234
ntth3	CoDom	0.00	1.59	2.21	0.56	0.001	1443.1		
ntth4	Dom	-0.01	[-0.01]	1.66	0.55	0.094	N/A		

ttdt1	Dom	-0.06	[-0.06]	1.85	0.61	0.206	N/A		
ttdt2	Dom	-0.03	[-0.03]	2.25	0.74	0.134	1997.5	12.96	0.003180
ttdt2	CoDom	-0.01	2.91	3.50	0.77	0.004	2010.4		
ttdt3	Dom	-0.05	[-0.05]	2.25	0.86	0.162	N/A		
ttdt4	Dom	-0.01	[2.73]	2.73	1.02	0.003	N/A		

ttth1	Dom	-0.05	[-0.05]	1.14	0.48	0.218	N/A		
ttth2	Dom	0.00	[2.12]	2.12	0.76	0.002	N/A		
ttth3	Dom	-0.03	[-0.03]	1.67	0.74	0.132	N/A		
ttth4	Dom	-0.02	[-0.02]	1.99	0.65	0.106	N/A		

^a^m(AA) = trait mean value of genotype AA

^b^f(a) = frequency of allele a

^c^[ ] = parameter fixed to equal homozygote value

^d^The chi-square and *p*-values are for the co-dominant (CoDom) vs. dominant (Dom) model comparisons, when the co-dominant model could be fit

^e^N/A = -2lnLIKE not presented because no comparison was possible

**Table 2:** Four regions with LOD score > 2.0.

			**Modeled, 2-marker (LINKAGE)**		**Model free, multipoint (MERLIN)**	
				
**Chromosome**	**Markers**	**Adjusted trait**	**LOD**	**p(LOD)**	**KC-LOD**	**p(KC)**
3	D3S2406-GATA128C02	ttdt1	2.01	0.0024	0.68	0.04
	GATA128C02-D3S2459	ttdt1	1.34	0.0130	"	"

4	D4S1558-D4S2361	ecb21	2.08	0.0020	0.29	0.12
	D4S2361-D4S1559	ecb21	1.08	0.0260	0.11	0.20

9	GATA175H06-D9S925	ttth1	2.30	0.0011	0.63	0.04
	D9S925-D9S304	ttth1	1.40	0.0113	"	"

12	D12S1090-D12S390	ecb21	2.61	0.0005	0.90	0.02
	D12S390-D12S398	ecb21	2.17	0.0016	"	"
